# Matrix regulation of idiopathic pulmonary fibrosis: the role of enzymes

**DOI:** 10.1186/1755-1536-6-20

**Published:** 2013-11-26

**Authors:** Deborah L Clarke, Alan M Carruthers, Tomas Mustelin, Lynne A Murray

**Affiliations:** 1 MedImmune Ltd, Granta Park, Cambridge CB21 6GH, UK; 2 MedImmune LLC, Gaithersburg, MD, USA

**Keywords:** Fibrosis, Matrix, Chronic remodeling, IPF

## Abstract

Repairing damaged tissues is an essential homeostatic mechanism that enables clearance of dead or damaged cells after injury, and the maintenance of tissue integrity. However, exaggeration of this process in the lung can lead to the development of fibrotic scar tissue. This is characterized by excessive accumulation of extracellular matrix (ECM) components such as fibronectin, proteoglycans, hyaluronic acid, and interstitial collagens. After tissue injury, or a breakdown of tissue integrity, a cascade of events unfolds to maintain normal tissue homeostasis. Inflammatory mediators are released from injured epithelium, leading to both platelet activation and inflammatory cell migration. Inflammatory cells are capable of releasing multiple pro-inflammatory and fibrogenic mediators such as transforming growth factor (TGF)β and interleukin (IL)-13, which can trigger myofibroblast proliferation and recruitment. The myofibroblast population is also expanded as a result of epithelial cells undergoing epithelial-to-mesenchymal transition and of the activation of resident fibroblasts, leading to ECM deposition and tissue remodeling. In the healthy lung, wound healing then proceeds to restore the normal architecture of the lung; however, fibrosis can develop when the wound is severe, the tissue injury persists, or the repair process becomes dysregulated. Understanding the processes regulating aberrant wound healing and the matrix in the chronic fibrotic lung disease idiopathic pulmonary fibrosis (IPF), is key to identifying new treatments for this chronic debilitating disease. This review focuses primarily on the emerging role of enzymes in the lungs of patients with IPF. Elevated expression of a number of enzymes that can directly modulate the ECM has been reported, and recent data indicates that modulating the activity of these enzymes can have a downstream effect on fibrotic tissue remodeling.

## Review

### Fibrotic matrix versus normal matrix

**Figure 1:**
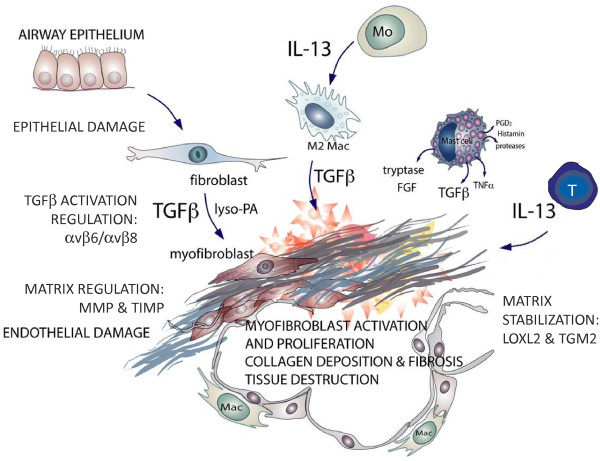
**Schematic outlining the key effector cells that generate transforming growth factor (TGF)β and interleukin (IL)-13 and the enzymes associated with idiopathic pulmonary fibrosis (IPF).** Multiple cell types are found at sites of lung fibrosis. Many are direct producers of extracellular matrix (ECM), or indirectly promote the generation and deposition of aberrant matrix.

Multiple mechanisms and mediators contribute to an altered ECM in IPF. Components of the ECM are produced intracellularly by resident cells and secreted into the ECM where they aggregate with the existing matrix, and can exert a powerful influence over cell functions. The ECM is composed of an interlocking mesh of fibrous proteins and glycosaminoglycans, but the most abundant ECM component in most tissues is collagen. The principal collagens are the interstitial types I and III, which serve to form a fibrous network in the interstitium of tissues, and these are elevated in the parenchyma of patients with IPF [1]. Type IV collagen is the major component of the basement membrane. This matrix dysregulation and deposition in the lungs of patients with asthma can lead to a thickening in the basement membrane and directly contribute to disease via alterations in lung function [2]. In IPF, increased matrix leads to impaired gaseous exchange, and matrix components such as hyaluronic acid can further exacerbate the inflammatory milieu seen in the lung and contribute to disease progression [3] (Figure 1).

### Matrix-producing cells: fibroblasts and myofibroblasts

The aberrant collagen deposition in the lungs of patients with IPF derives predominantly from activated fibroblasts and myofibroblasts. These cells are classically thought to derive from post-embryonic lung fibroblasts. However, accumulating data support a bone marrow-derived source for these cells, called fibrocytes. In experimental models, fibrocytes contributed to fibrotic remodeling both directly via ECM production and indirectly through the paracrine regulation of other fibrogenic cells [4,5]. Fibroblasts may also derive from epithelial-to-mesenchymal transition [6] or endothelial-to-mesenchymal transition [7], although their precise contribution to α-smooth muscle actin (α-SMA)-expressing cells remains controversial [8], and the contribution to disease progression in IPF is not firmly established.

Fibroblasts play central roles both in the maintenance of normal tissue function and in the wound healing response. The lung is a dynamic organ, often under varying degrees of motility and stress. By generating ECM, fibroblasts provide a scaffold for cells and a sink for mediators to be able to respond to the rapid changes in shear force. In the lung, fibroblasts are found in greatest numbers in the subepithelial layer of the conducting airways and the interstitium of the lung parenchyma, which puts them in a prime location to interact with the epithelial and endothelial cells.

Fibroblasts and myofibroblasts are metabolically active cells, capable of synthesizing, secreting, and degrading ECM components, including collagens, proteoglycans, tenascin, laminin, and fibronectin. The secretion of ECM proteins is closesly regulated. Moreover, fibroblasts generate matrix metalloproteinases (MMPs) and their inhibitors, tissue inhibitors of metalloproteinases (TIMPs), thus controlling tissue architecture and matrix turnover rates, and contributing to both the initiation and resolution phases following injury. The added contractile properties of myofibroblasts are also central to controlling wound healing as well as tissue architecture [9]. *In vitro*, fibroblast-to-myofibroblast trans-differentiation can be induced by transforming growth factor (TGF)β1 and it has been hypothesized that TGFβ1 found locally at sites of fibrosis trans-differentiate resident fibroblasts into myofibroblasts [10,11]. Myofibroblasts are generally absent in lung parenchyma; however, in IPF, one of the hallmarks of disease is the presence of α-SMA-positive cells in and around fibroblastic foci.

IL-13 is another pro-fibrotic mediator that can elicit a number of fibroblast responses *in vitro*, as well as directly promoting fibrosis *in vivo*. Fibroblasts isolated from the lungs of patients with IPF exhibit an increase in IL-13 receptor expression for both IL-13Rα1 and IL-13Rα2 [12,13]. Moreover, IPF fibroblasts are hyper-responsive to IL-13 stimulation *in vitro*, resulting in enhanced collagen production, differentiation to of fibroblasts to myofibroblasts, and increased TGFβ expression [13]. Both T cells and alternatively activated macrophages express IL-13 in the IPF lung [14]. Both of these cell types are found in increased numbers in IPF [15,16]. *In vivo* mouse models have shown a pro-fibrotic role for IL-13Rα2 [17], classically thought of as a decoy receptor for IL-13. However, the relevance of this to human disease still needs to be determined.

Interestingly, fibroblasts isolated from fibrotic lung tissue are phenotypically different to non-fibrotic fibroblasts [18-20]. Fibrotic fibroblasts exhibit altered responsiveness to growth factors and also enhanced chemokine receptor expression, suggesting a lower tolerance to exogenous stimuli. The progression and severity of IPF has also been closely associated with regions of fibroblast accumulation and proliferation, to the extent that these regions have become a reliable indicator of survival [18-20]. The increased number of collagen-producing mesenchymal cells seen in these diseases suggests that these cells are either hyperproliferative and/or resistant to apoptosis, and both of these alterations have been observed *in vitro*[18,19,21-23]. Collectively, IPF fibroblasts behave more like tumor cells, indicating that they have undergone epigenetic changes, or have transformed somehow from a normal lung fibroblast to a disease phenotype.

### Remodeling of the matrix and control of matrix turnover

The turnover rates of matrix molecules and the proteases that degrade them are under control of an extensive network of cytokines, growth factors, proteases, lipid mediators, and mechanical forces. Fibroblasts synthesize a host of matrix components driving matrix deposition, and an abundant number of enzymes regulate matrix degradation including MMPs and serine proteases [24]. Collagen is also degraded both intracellularly and extracellularly, and the maintenance of tissue homeostasis is dependent on the regulation of matrix production and degradation within the tissue. In diseased tissue, it is clear that this finely balanced process is dysregulated, leading to tissue destruction and excessive matrix deposition. Mediators involved in this process leading to tissue fibrosis include locally released polypeptide growth factors (such as platelet-derived growth factor, epidermal growth factor, fibroblast growth factor) cytokines such as TGFβ and the interleukins, and cellular enzymes, which will be discussed below [25].

MMPs exert proteolytic activities on various proteins including many ECM components, and are thus central to ECM formation [26]. Numerous cell types in the lung are capable of expressing MMPs, including epithelium, fibroblasts, myofibroblasts, and macrophages. These cells have been shown to be elevated in asthma and chronic obstructive pulmonary disease (COPD) [26], as well as in IPF, where MMP1, MMP2 and MMP9 were shown to be co-localized to the epithelium surrounding fibrotic lesions, while increased TIMP2, was also observed suggesting that the MMP activity may be inhibited and that the fibrotic region is not degraded [27]. Other work has reported a role for MMP3, which is elevated in the IPF lung [28]. Overexpression of MMP3 leads to pulmonary fibrosis in the rat lung, while mice lacking MMP3 are protected [28]. *In vitro* work suggests a role for MMP3 in the activation of β-catenin signaling and the induction of epithelial-to-mesenchymal transition, a process thought to contribute to the pathogenesis of IPF [29]. IL-13 can directly induce a number of pro-fibrotic MMPs, including MMP9 and MMP12 [30], and MMP7 [31], all of which are elevated in the lungs of patients with IPF. In addition to MMP3, pro-MMP7 is also elevated in the bronchoalveolar lavage fluid of patients with IPF, and is thought to be produced by the hyperplastic alveolar and metaplastic bronchiolar epithelial cells, and activated locally in the lung [32]. Membrane type-1 MMP (MT1-MMP or MMP14) has also been reported to serve as a key effector of type I collagenolytic activity in pulmonary fibroblasts [33]. Other MMPs have been associated with the genetic risk of IPF. Polymorphisms of the MMP-1 promoter have been shown to potentially confer increased risk for IPF [34]. High-resolution computed tomography (HRCT) and histopathologic evaluation of fibrosis and tissue destruction in IPF have been associated with pulmonary emphysema, with expression of MMP2, MMP7, MMP9, and MT1-MMP by fibroblasts of myofibroblastic foci being predominant in fibrosis [35].

### Other protease activity in IPF

In addition to MMP dysregulation, other enzymes are known to play a role in IPF. Coagulation proteases, besides their important role in fibrin formation, are now well recognized to exert pro-fibrotic cellular effects via activation of protease-activated receptors (PARs) [36]. Mast cells co-cultured with lung fibroblasts become activated and release tryptase, which in turn promotes lung fibroblast proliferation via PAR-2 [36]. Tryptase-mediated stimulation of fibroblast proliferation occurs via activation of the protease-activated receptor PAR-2 [37], and more recently a PAR-2 dependency has also been demonstrated for tryptase induction of collagen and fibronectin synthesis by fibroblasts [38]. Furthermore, those authors hypothesized that the increase in PAR-2 expression observed in lung fibroblasts from patients with IPF could sensitize these cells to the effects of mast cell-derived tryptase [36,38]. PAR-2 is elevated in IPF lungs, and is upregulated by TGFβ in lung fibroblasts [36]. PAR-2 has also recently been reported to play a role in aggravating pulmonary fibrosis. An absence of PAR-1 signaling in PAR-1 knockout mice conferred protection in a bleomycin model [39], and thrombin stimulation of proteoglycan expression is mediated by PAR-1 activation of TGFβ1RI [40]. More recently, a PAR-1 antagonist attenuated the expression of TGFβ1-promoted airway remodeling in ovalbumin-allergic rats [41]. PAR-2 has also recently been reported to play a role in aggravating pulmonary fibrosis. Mast cells co-cultured with lung fibroblasts become activated, and release tryptase, which in turn promotes lung fibroblast proliferation via PAR-2 [38].

### Enzymes affecting matrix cross-linking

Enzymes such as transglutaminases (TGs) and lysyl oxidases (LOXs) are also reported to contribute to the pathogenesis of fibrosis through modification of the ECM. TGs are a family of nine enzymes that catalyze post-translational bonds between proteins via mechanism such as transamidation [42]. TG2 (also known as tissue transglutaminase) is the most abundant and widely expressed member of the TG family, being present in many cell types including fibroblasts, macrophages, smooth muscle cells, hepatocytes, red blood cells, cardiac myocytes, neurons, chondrocytes, and kidney cells [43]. TG2 is reported to promote tissue fibrosis through cross-linking extracellular collagen and fibronectin, making them more resistant to breakdown [44], but is known to exert multiple other functions that may promote fibrosis [45-49]. TG2 has been shown to promote liver and kidney fibrosis, and to be elevated in patients with kidney disease [50-54]. TG activity was reportedly increased in a rat model of pulmonary fibrosis [55]. In a mouse model of lung injury induced by bleomycin, bleomycin-induced epithelial damage was mediated through TG2, resulting in IL-6 release and the differentiation of IL-17-producing T cells, with subsequent inflammatory amplification in the lung [56]. In addition, the authors reported that fibroblast-derived TG2, acting downstream of TGFβ, was also important in the effector phase of fibrogenesis [56]. TG2 knockout mice were protected from bleomycin-induced lung fibrosis, and TG2 expression and activity were increased in patients with IPF versus normal controls [57].

The LOX family is a family of five enzymes that facilitate the covalent cross-linking of type I collagens via catalysis of the oxidative deamination reaction between a lysyl or a hydroxylysyl residue [58,59]. LOXL1 regulates collagen cross-linking and total collagen levels in angiotensin II-induced hypertension in rodents [60], has been associated with renal fibrosis [61], and when overexpressed, induces cardiac hypertrophy in mice [62]. LOXL2 was reported to increase collagen accumulation in breast and glioma tumors [63,64], and has been associated with liver fibrosis [65], as well as IPF [64]. Treatment of mice with an anti-LOXL2 monoclonal antibody reduced the fibrotic burden in a model of cancer and lung fibrosis, in part via reduction in disease-associated fibroblasts. Anti-LOXL2 also led to reduced production of growth factors and cross-linked collagenous matrix, and also reduced TGFβ signaling, and thus those authors postulated that LOXL2 could potentially mediate fibroblast activation *in vivo* through its enzymatically catalyzed cross-linking of fibrillar collagen and corresponding increase in local matrix tension, resulting in activation of TGFβ1 signaling from the latent complex [64]. Targeting the cross-linking of collagen is an attractive therapeutic angle for patients with fibrosis, and indeed, agents blocking LOXL2 are now entering clinical trials in patients with IPF.

### The role of the matrix in promoting fibroblast activation and inflammation

The ECM, as discussed, is a complex mixture of proteins, proteoglycans, and glycosaminoglycans, which supports cell and tissue architecture. However, the ECM can also alter cell adhesion, migration, proliferation/survival, and differentiation. Recent data have indicated that increased matrix stiffness increases fibroblast proliferation and contractility [66]. Fibrotic matrix is stiffer than normal lung matrix (6 to 20 kPa versus 1 kPa) [67]. Moreover, normal fibroblasts exhibit IPF cell properties when they are subjected to matrices with heightened stiffness, including increased differentiation to myofibroblasts, along with increased proliferation and resistance to apoptosis [67-70]. Another key pro-fibrotic role for increased matrix stiffness is the promotion of latent TGFβ activation, whereas compliant matrices have lower levels of TGFβ activation [68]. Previous studies comparing normal and IPF fibroblasts have been conducted on glass or tissue culture plastic, and some of the underlying differences in cell responses may actually be due to the different types of matrix produced by the cells on different substrates. Therefore, future studies profiling discrete differences in matrix components are warranted.

ECM can also directly promote inflammation. Components of the ECM, namely hyaluronan and elastin, are reported to exhibit chemotactic activity on inflammatory cells, enhance phagocytic function, enhance adhesion of polymorphonuclear neutrophils (PMNs), induce immune responses, and change gene expression profiles in inflammatory cells [71]. In the initial inflammatory phase of wound repair, the glycosaminoglycan hyaluronic acid is abundant, and acts as a promoter of early inflammation. However, failure to remove ECM degradation products from the site of tissue injury contributes to the unremitting inflammation and destruction observed in IPF. Clearance of hyaluronan fragments is thought to be dependent both on its receptor CD44 and on recognition by the host via Toll-like receptor (TLR)2 and TLR4 [3]. Hyaluronan-TLR2 and hyaluronan-TLR4 signals regulate both the innate inflammatory response as well as the epithelial cell integrity that is crucial for recovery from acute lung injury [3].

The ECM also plays a role in leukocyte adhesion. Adhesion of PMNs to ECM proteins has been shown to be important for their migration, and is thought to participate in PMN recruitment to sites of inflammation. Migration of PMNs through the parenchyma can be influenced by the composition of the ECM [72], with multiple studies demonstrating increased adherence of PMNs to surfaces coated with fibronectin and collagen [73]. ECM components such as fibronectin can also act as chemotactic agents for PMNs, suggesting that local rates of migration within tissues are potentially regulated by several different ECM components. Adhesion of PMNs to collagens is thought to be mediated via the integrin CD11b/CD18, found on PMNs [74], as shown by antibody-blocking experiments.

### Matrix signaling: growth factors and integrins

It is now over three decades since the discovery of TGFβ [75], and its pleiotropic effects on cell growth, immune function, and proliferation have been well documented. Acting as a key pro-fibrotic molecule, TGFβ is a central stimulator of collagen production in the pathogenesis of pulmonary fibrosis [76]. The three mammalian isoforms of the TGFβ family, TGFβ1, TGFβ2, and TGFβ3 are secreted as pro-TGFβ forms, covalently bound to a pro-peptide (latent associated protein (LAP)β1, LAPβ2, and LAPβ3) in a complex referred to as the small latent complex (SLC). These structures prevent the engagement and agonism of the active molecule at TGFβ receptors. These complexes are targeted to structures within the ECM through latent TGF binding proteins (LTBPs) [77] by association with other ECM proteins such as fibronectin, fibrillin-1, and vitronectin. The majority of cells secrete TGF as an SLC with LTBP-1 [78,79]. Thus, the large latent complex represents a storage depot of latent TGFβ for activation. Disruption of this process allows the release of TGFβ1, in a process known as TGFβ1 activation.

Activation of latent TGFβ can occur by multiple mechanisms, and is normally associated with proteolytic degradation of the LAP or an alteration of LAP confirmation to allow the activation of TGFβ; for example, bone morphogenetic protein-1, multiple MMPs, plasmin, urokinase-type plasminogen activator (uPA), tissue-type plasminogen activator, and thrombin [80]. Among proteolytic enzymes, uPA-mediated activation of plasmin is involved in the activation of latent TGFβ1 by proteolytic cleavage within the N-terminal region of LAP [81]. Binding of uPA to its receptor uPAR can trigger intracellular signal transduction via interaction with the α5β1 integrin [82], in addition to focusing plasmin production to release ECM factors and activate latent TGFβ1 [83,84]. The transmembrane αv integrins have been shown to bind to and activate latent TGFβ1 and β3 by binding to arginine-glycine-aspartic acid (RGD) motifs. This RGD motif is absent in the LAP of TGFβ2, and consequently no integrin-mediated activation of TGFβ2 has been described to date. All the αv integrins, αvβ1, αvβ3, αvβ5, αvβ6, and αvβ8, and the integrins α5β1 and α8β1 bind in this way.

Evidence for this major role for an integrin in TGFβ activation was first highlighted using β6-deficient mice that are unable to form the αvβ6 integrin [85] These mice exhibited an exaggerated inflammatory response similar to that seen with TGFβ1 and Smad3 knockout mice [86]. Subsequently, direct evidence of TGF activation *in vivo* was demonstrated for αvβ5 [68], αvβ6 [85,] and αvβ8 [87].

The integrins αvβ3, αvβ5, αvβ6, and αvβ8 have been shown to activate latent TGFβ1 via two distinct modes of action [88]. In the case of αvβ8, which is expressed on normal epithelial cells, fetal fibroblasts, and dendritic and neuronal cells [87], it binds with high affinity to latent TGFβ1 by recognition of the RGD domain, and activation is mediated by anchoring of the molecule and its close proximity with MMP14, bringing about its proteolytic cleavage [89] Fibroblast-specific inducible β8 silencing reduced lung remodeling and TGFβ signaling in a chronic allergen challenge model [90], demonstrating a key *in vivo* function of this integrin.

In the case of αvβ3, αvβ5, and αvβ6, these integrins have been shown to activate latent TGFβ1 in a proteolysis-independent manner, by transmission of contractile cell forces within the LLC. These forces result in deformation of the latent complex to liberate active TGFβ1 [68,91]. The importance of the role of LTBP-1 as a lever to decrease the threshold for TGFβ1 activation has recently been highlighted [92].

### Current clinical approaches measuring and targeting the matrix

Despite our increasing understanding of the pathology driving IPF, and the key mechanisms involved in this disease, another area receiving much interest is how to measure matrix and collagen turnover clinically in patients, either to enable identification or to determine disease progression. HRCT can be used to clinically diagnose and determine the extent of lung fibrosis; however, repeated HRCT exposure to a patient who already has lung fibrosis may not be clinically feasible in the long term. Recently proteolytic generation of pathological and tissue-specific fragments of proteins has received increased attention [93] as a method of identifying potential markers of chronic lung disease in which matrix dysregulation is a key feature, such as COPD and IPF.

It has been reported that these protein fragments (also known as neoepitopes or protein fingerprints) are more accurate predictors than the unmodified intact proteins [93]. Examples include a type III collagen fragment generated by MMPs as a marker for generalized and liver fibrosis [92,94], a type II collagen degradation product generated by MMPs for osteoarthritis and rheumatoid arthritis [95], and a type I collagen fragment generated by cathepsin K as a diagnostic tool for measuring and monitoring bone resorption [96]. MMP9 and MMP12 have been associated with elastin degradation, and are implicated in chronic respiratory diseases such as IPF. Recent work in this field identified an elastin fragment, ELN-441, which is released by the action of MMP9 and MMP12 on elastin. This fragment was elevated in the serum of patients with IPF or COPD, and may represent a novel method for detecting ECM in IPF and other chronic lung diseases, following further clinical validation [97].

Another approach that is under development is using stable isotope labeling techniques in combination with ultra-high-resolution mass spectrometry. A recent publication demonstrated the potential of monitoring collagen turnover *in vivo* using the intratracheal bleomycin mouse model [98]. Mice were challenged with bleomycin, and given deuterated drinking water 1 week prior to terminal endpoint, and the percentage of hydroxyproline that contained deuterateswater indicated the amount of new collagen [98]. The results indicated that new collagen generation closely mirrored cell proliferation, and the initial uptake of deuterated water peaked as early as 1 week post-bleomycin, when it subsequently declined, whereas histological fibrosis also peaked 1 week post-bleomycin but remained elevated throughout the remaining 5 weeks [98]. Collectively, these results indicated that the fibrosis observed in this model was initially induced by new collagen deposition and that the continued pathology was not due to constant collagen turnover, but more to maintenance of the synthesized ECM. Although the intratracheal bleomycin model has significant limitations and does not closely resemble clinical IPF, the model has provided insights in to some of the key pathways associated with IPF. However, if this phenomenon, of an initial burst of ECM generation that is then maintained resulting in chronic lung fibrosis, applies to IPF, future anti-fibrotic therapies should aim to target the maintenance of collagen and not the initial synthesis.

## Conclusions

As described in this review, the matrix provides a number of pro-fibrotic mechanisms, including direct physiochemical activating properties, a sink for pro-fibrotic and pro-inflammatory mediators, and fostering of ECM-producing cell survival. Therefore, disrupting the aberrant matrix that has accumulated in the IPF lung may result in a number of pathways being inhibited, and might provide significant improvements in gas exchange in the lung, resulting in considerable improvement in patient mortality and morbidity.

## Abbreviations

BMP: Bone morphogenic protein; COPD: Chronic obstructive pulmonary disease; ECM: Extracellular matrix; HRCT: High-resolution computed tomography; IPF: Idiopathic pulmonary fibrosis; LOX: Lysyl oxidase; LTBP: Latent transforming growth factor beta binding protein; MMP: Matrix metalloproteinase; PAR: Protease-activated receptor; PMN: Polymorphonuclear neutrophil; SMA: Smooth muscle actin; TG: Transglutaminase; TGF: Transforming growth factor; TIMP: Tissue inhibitor of matrix metalloproteinases.

## Competing interests

The authors declare that they have no competing interests.

## Authors’ contributions

All authors contributed to the writing of this review article and have read and approved the final manuscript.
